# Metabolic Syndrome in Affective Disorders: Associations with Dark Triad Personality Traits

**DOI:** 10.3390/metabo13080956

**Published:** 2023-08-18

**Authors:** Fiona Brugger, Elena M. D. Schönthaler, Andreas Baranyi, Eva Z. Reininghaus, Dirk von Lewinski, Nina Dalkner

**Affiliations:** 1 Department of Psychiatry and Psychotherapeutic Medicine, Medical University Graz, 8036 Graz, Austria; 2 Department of Cardiology, Medical University of Graz, 8036 Graz, Austria; dirk.von-lewinski@medunigraz.at

**Keywords:** metabolic syndrome, Body Mass Index, blood pressure, Dark Triad, affective disorders

## Abstract

Previous research has focused on the relationship between affective disorders (AD) and metabolic syndrome (MetS). Aside from biological and lifestyle factors, personality traits were identified as influencing aspects. In particular, the Dark Triad personality traits (DT; Machiavellianism, narcissism, psychopathy) were connected to both AD and worse somatic health, thus possibly resulting in MetS. This observational study aimed to investigate the associations between DT and anthropometric parameters and differences in the DT traits concerning the presence of MetS in individuals with AD. A total of 112 individuals (females = 59, males = 51, diverse = 2, *M*_age_ = 47.5, *SD*_age_ = 11.5) with AD filled out the Short Dark Triad questionnaire. Body Mass Index (BMI) and MetS criteria, including blood pressure, waist circumference, lipid, and glucose levels, were assessed. For Machiavellianism, a positive association with BMI (*r* = 0.29, *p* < 0.05) and a negative association with systolic blood pressure (*r* = −0.23, *p* < 0.05) were found. No relationship between the overall MetS and DT score (*r* = 0.08, *p* = 0.409) was observed. The results were limited by the lack of a control group and the cross-sectional study design, which does not allow for the determination of causality. Machiavellianism was associated with a higher BMI and lower systolic blood pressure, indicating a deteriorating health effect of this trait. Possibly, the higher prevalence of MetS in AD stems from aspects such as lifestyle or medication intake, which might also be influenced by DT. Further research is needed to disentangle underlying mechanisms.

## 1. Introduction

Affective disorders (AD), including unipolar and bipolar disorders, are mainly characterized by episodes of mood disturbances, and altered activity levels. Compared to mentally healthy individuals, individuals with AD tend to have a lower life expectancy [1,2], which is reattracted to increased suicidality and low socioeconomic status [3,4] but also to a higher prevalence of somatic comorbidities. Along with cardiovascular diseases, metabolic syndrome (MetS) is one of the most common somatic problems in AD [5].

MetS is a cardiovascular risk cluster consisting of truncal obesity and additional factors such as glucose tolerance disorder, or type 2 diabetes mellitus [6]. MetS shows a positive association with BMI [7]. Recent research indicated that individuals with AD have an increased risk of developing MetS compared to mentally healthy individuals (e.g., [8]). Major risk factors for developing MetS are lifestyle habits and deteriorated health behavior (e.g., poor diet). Indeed, individuals with AD have a higher frequency of smoking, poor nutrition, and lack of physical activity compared to mentally healthy individuals [9], which partially explains the higher prevalence of MetS in this population. Further, other studies examining the relationship between AD and MetS found that MetS and lifestyle factors increase the risk of developing other impairments, such as a deterioration in cognitive functions (e.g., [10]). Additionally, mood-stabilizing medication has metabolic effects and can contribute to weight gain and the development of MetS [11]. Considering these findings, it is necessary to understand the factors driving the relationship between AD and MetS to prevent further somatic comorbidities in an already vulnerable population.

Several studies demonstrated that personality factors play a decisive role in the development of MetS (e.g., [12,13]) since personality traits influence health behaviors and, thus, possibly the development of MetS. For example, out of the Big Five personality traits, higher agreeableness and conscientiousness, as well as lower neuroticism, were shown to promote positive health behaviors [14]. Moreover, higher extraversion, agreeableness, and neuroticism, as well as lower conscientiousness, were associated with obesity [15], which is indicative of metabolic disturbances. Aside from health-promotive personality traits, there are other personality traits, such as the Dark Triad personality traits, which are known to have a detrimental effect on health behaviors. Such traits, however, have been rarely researched in the context of obesity or MetS.

The Dark Triad personality traits (DT), including Machiavellianism, narcissism, and psychopathy, are overlapping traits that manifest to an individual extent in each person [16]. Callousness, manipulation, disagreeableness, and a lack of empathy comprise the common core of the DT. While individuals scoring high in narcissism and psychopathy tend to show more self-centered behavior, individuals high in Machiavellianism are more strategically involved in manipulating others to achieve their goals. More specifically, Machiavellianism encompasses cynical behaviors, callousness, disagreeableness, manipulativeness, and a lack of moral standards, emotional bonds, and interpersonal understanding. Psychopathy entails interpersonal manipulation, callous emotionality, a risk-seeking lifestyle, and a pronounced lack of guilt, empathy, or remorse. Individuals high in narcissism are strongly self-deceptive and demonstrate a greater need for admiration, vanity, arrogance, grandiosity, and, subsequently, emotional instability [17,18,19,20,21]. DT have been shown to worsen health behavior, resulting in poor diet, lack of physical activity, low sleep quality, and mental health problems [22]. This was also evident in the recent COVID-19 pandemic, in which the DT were found to result in less compliance to prevention guidelines due to a lack of fear [18] and maladaptive health beliefs [23], which might lead to less frequent health behaviors and worse general health. Further, the DT were connected to metabolic disturbances. For instance, Machiavellianism and psychopathy were found to be associated with higher cholesterol and blood pressure and a higher prevalence of diabetes mellitus and obesity. In contrast, narcissism was shown to be negatively associated with most metabolic parameters and may, thus, even have a protective effect on somatic health [24].

Relatedly, the DT were not only found to be associated with health behaviors and metabolic alterations and, thus, possibly with the development of MetS but were also associated with the development of AD (e.g., [25]). For example, individuals high in psychopathy were found to be more likely to meet the criteria for depression [24]. On the contrary, other studies revealed that psychopathic traits represent a protective factor in the development of depressive symptoms due to their association with lower stress levels (e.g., [26]). Machiavellianism was previously positively associated with depression (e.g., [27]). On the contrary, grandiose narcissism has been identified as a protective factor in the development of depressive symptoms [28].

Since DT are connected to AD, health behaviors, and metabolic parameters, the question remains whether there is an association between DT, AD, and MetS altogether. Based on the literature described above, the existing associations between DT and AD [24,25,26,27,28], DT and health behaviors [18,22,23], and DT and metabolic health [24] suggest that there might be an association between DT and metabolic parameters in individuals with AD. This current study, thus, examined whether there is a relationship between DT and anthropometric data, including the measurement of MetS in a sample of individuals with AD. It was hypothesized that (1) there is a significant positive relationship between the DT and MetS and that (2) there is a significant difference in the individual manifestation of single DT traits (Machiavellianism, narcissism, and psychopathy) depending on the presence of MetS in individuals with AD. Finally, it was assumed that (3) there are positive significant associations between the single DT traits, the single MetS parameters (e.g., cholesterol), and BMI in individuals with AD.

## 2. Materials and Methods

### 2.1. Sample

For this study, 203 psychiatric inpatients with AD (diagnosis according to the International Classification of Diseases (ICD-10) [29]) were recruited at the Department of Psychiatry and Psychotherapeutic Medicine in Graz between November 2021 and July 2022. Participants were invited to this study if they fulfilled the inclusion criteria of having an AD and being of legal age (≥18 years). Thus, we applied a clinical sampling method. Subsequently, 56 participants without MetS were matched to 56 participants with MetS according to sex (same sex) and age (same age +/− 3 years). A total of 91 participants without MetS could not be matched and were thus excluded from data analyses. However, the analyses were also conducted once with all participants (under covariate adjustment for sex and age), obtaining the same results as described below (see Supplementary Material for results on the full sample (*n* = 203); Tables S1 and S2). After matching the participants, we examined data of 112 individuals (females = 59, males = 49, other = 4; *M*_age_ = 47.6, *SD*_age_ = 11.5). An a priori power analysis was conducted with G*Power (version 3.1), which indicated that at an α-level of 5%, a power of 80%, and an assumed medium effect size (*r* = 0.3), the minimum sample size is *n* = 82, which is well below the collected sample size [30]. All participants gave written informed consent prior to participating in this study. This study was approved by the Ethics Committee of the Medical University of Graz (EC-number: 33–632 ex 20/21) and was conducted in accordance with the Declaration of Helsinki. This study was preregistered at AsPredicted.org (https://aspredicted.org/F8T_RW8, accessed on 17 August 2023).

### 2.2. Materials

All questionnaires were presented in German with the online survey tool LimeSurvey (Version 3.40). This study was part of a large-scale ongoing study on health behaviors and DT in individuals with AD and mentally healthy individuals.

#### 2.2.1. Sociodemographic Data

We assessed sex, age, height, weight, educational background, relationship status, residence, employment, somatic and psychiatric diagnoses, and somatic and psychiatric medication of all participants.

#### 2.2.2. Dark Triad

The Short Dark Triad (SD3; [31]) is a self-assessment questionnaire examining the personality traits of the DT with 27 items on three scales (Machiavellianism, narcissism, psychopathy). Participants were asked to rate all presented statements on an ascending five-point Likert scale, ranging from (1) = “not at all” to (5) = “completely”. Scores for the single DT scales and the total DT score were built by calculating the mean of the corresponding items. All scales indicated sufficient internal consistency (as indicated by Cronbach’s α; Machiavellianism: α = 0.74, narcissism: α = 0.60, psychopathy: α = 0.59). We excluded one psychopathy item due to deterioration of internal consistency, leading to a Cronbach’s alpha of α = 0.65 on the psychopathy scale. Notably, Cronbach’s α was lower in narcissism and psychopathy. However, these values are consistent with previous studies using the SD3 (e.g., [32]).

#### 2.2.3. Metabolic Syndrome (MetS)

To assess the presence of MetS, we collected data on the criteria as proposed by the International Diabetes Federation [6]. To qualify for MetS, the main criterion (i.e., waist circumference: women ≥ 80 cm, men ≥ 94 cm for Western Europe) and at least two secondary criteria must be fulfilled (i.e., serum triglyceride levels (≥150 mg/dL), high-density lipoprotein cholesterol levels (women < 50 mg/dL, men < 40 mg/dL), blood pressure (systolic ≥ 130 mmHg, diastolic ≥ 85 mmHg), fasting plasma glucose levels (≥100 mg/dL)). If participants had diabetes, hyperlipidemia, or hypertension and received pharmacological treatment for these issues, they also met the corresponding criterion [33]. Waist circumference was measured slightly above the hipbones using a tape measure, and blood pressure was recorded with a blood pressure device. Laboratory parameters (such as high-density lipoprotein cholesterol levels) were collected within the routine examinations conducted in the clinic.

#### 2.2.4. Obesity

Indicative of obesity, the BMI was included as secondary outcome parameter. BMI was defined using the criteria of the International World Health Organization as follows [34]:

*BMI* = (*person’ s weight* [kg])/(*square of the person’s height* [m^2^])

### 2.3. Statistics

First, a dichotomous variable was formed using all the parameters for diagnosing MetS, providing information on the (non-)presence of the syndrome. To calculate the relationship between MetS and the DT, the association of the dichotomous MetS variable with the DT was examined using point-biserial bivariate Pearson correlations. To conduct these correlations, subjects who identified themselves as diverse and their matches were excluded from analyses, resulting in a sample size of *n* = 108. Moreover, we computed a binomial logistic regression analysis to further examine the relationship between MetS and the DT. Secondly, to examine the difference in the DT traits Machiavellianism, narcissism, and psychopathy depending on MetS, we conducted a multivariate analysis of variance (MANOVA). Since the previous correlation analyses indicated that sex had a significant influence on the DT (*r* = 0.32, *p* < 0.001), sex was included as a covariate in all further analyses. Hence, we ultimately conducted a multivariate analysis of covariance (MANCOVA) with the between factor MetS, the covariate sex, and the outcome parameters Machiavellianism, narcissism, and psychopathy. Finally, we administered a partial correlation analysis to determine the relationships between single MetS parameters (e.g., waist circumference), BMI, and the single DT traits, controlling for sex. For all correlation analyses, the Benjamini–Yekutieli adjustment was applied to adjust the α-level [35]. All hypotheses were tested two-sided (α = 0.05), and common statistical assumptions were met unless otherwise noted. As preregistered, outliers in personality traits were not excluded to maintain individual differences. Data and analysis scripts can be accessed at https://doi.org/10.17605/OSF.IO/U9KNS (accessed on 17 August 2023).

## 3. Results

All analyses were conducted using IBM SPSS Statistics 28 (IBM corp, Armonk, New York, NY, USA).

### 3.1. Descriptive Statistics

Table 1 shows the sample characteristics differentiated by MetS groups and significance tests to determine group differences (*χ*^2^-, *t*-tests, or Mann–Whitney *U*-test). A Mann–Whitney *U*-test indicated that individuals without MetS had higher educational degrees than individuals with MetS. Moreover, those with MetS reported significantly more somatic disorders than those without MetS. The BMI and the MetS parameters were significantly higher in the MetS group.

### 3.2. Associations and Differences in DT Regarding MetS

Correlation analyses indicated no significant associations between age, MetS, and the DT total score, respectively. However, sex was significantly positively correlated with both the DT total score and narcissism (see Table 2).

For the binomial logistic regression analysis, the single DT traits were entered as predictors and MetS as the dependent variable. The Hosmer–Lemeshow test indicated a good model fit (*χ*^2^(8) = 3.09, *p* = 0.928). The binomial logistic regression model was not statistically significant (*χ*^2^(3) = 5.50, *p* = 0.139, Nagelkerke’s *R*^2^ = 0.07). Neither narcissism (*b* = −0.60, *SE* = 0.37, *p* = 0.106; OR [0.27; 1.14] = 0.55) nor Machiavellianism (*b* = −0.11, *SE* = 0.29, *p* = 0.715; OR [0.63; 1.95] = 1.11), or psychopathy (*b* = −0.83, *SE* = 0.48, *p* = 0.081; OR [0.90; 5.87] = 0.55) predicted MetS. The overall percentage of accuracy in classification was 52.8%, with a sensitivity of 50% and a specificity of 55.6%.

Based on the abovementioned correlation results, sex was included as a covariate in the subsequent MANCOVA, which assessed the difference in the single DT traits depending on MetS. The results of the analysis indicated no significant difference between the MetS groups regarding Machiavellianism (*F*(1, 1.22) = 2.13, *p* = 0.148, η^2^*_p_* = 0.02), narcissism (*F*(1, 0.31) = 0.91, *p* = 0.344, η^2^*_p_* = 0.01), or psychopathy (*F*(1, 0.33) = 1.12, *p* = 0.277, η^2^*_p_* = 0.01).

### 3.3. Associations between the DT and Metabolic/MetS Parameters/Obesity

To examine the associations between single MetS parameters and the single DT traits while controlling for sex, a partial correlation analysis was conducted. Seven outliers deviating more than three *SD* were found in the parameters “triglycerides” and “plasma glucose” and were thus excluded, resulting in a sample size of *n* = 101. After applying Benjamini–Yekutieli adjustments, only the correlative relationships between Machiavellianism, BMI, and systolic blood pressure remained statistically significant (see Table 3).

## 4. Discussion

The aim of this study was to investigate whether there is an association between anthropometric measures, including BMI and MetS, and the DT in individuals with AD and whether there are differences in the single DT (Machiavellianism, narcissism, and psychopathy) depending on the presence of MetS. Moreover, we examined the associations between BMI, single MetS parameters, and single DT traits. Contrary to our hypothesis, our results showed no association between MetS and the total DT score. This was also reflected in further analyses, which revealed no significant difference in Machiavellianism, narcissism, or psychopathy, depending on the presence of MetS. However, we found that Machiavellianism was associated with lower systolic blood pressure and higher BMI, which is indicative of obesity.

Our finding of Machiavellianism being significantly associated with BMI and systolic blood pressure corroborates other studies, which showed a positive correlation between Machiavellianism and BMI [36], obesity, and high blood pressure (e.g., [22]), respectively. Notably, Machiavellianism was previously associated with worse health behavior in general [37]. This is explained by the fact that Machiavellianism promotes an unhealthy lifestyle, resulting in a worse diet and less physical activity due to the Machiavellian nature of planning thoughtfully, which causes stress to some extent as immediate needs are suppressed for long-term goals [24,38]. These factors could elevate the risk of metabolic diseases and result in a higher BMI and lower systolic blood pressure. However, when examining Machiavellianism in relation to MetS, no significant difference between those with and without MetS was found. In this population, metabolic disturbances could also stem from other illness-related factors, such as medication intake, unhealthy lifestyle, illness symptoms, or stigmatization, resulting in worse prevention and treatment of metabolic diseases.

Furthermore, no significant associations between narcissism, psychopathy, DT total score, and MetS criteria were found. Additionally, there were no significant differences in DT traits regarding MetS criteria, independently of sex. The lacking association between narcissism, psychopathy, and MetS criteria was also reflected by the non-significant associations between these traits and the single MetS parameters. Generally, these findings are not in line with the current literature. Recent studies point towards a positive effect of narcissism on health behavior, e.g., more physical activity and better dietary behavior due to narcissistic self-sufficiency or more participation in preventive health interventions. The health-promotive behavior in narcissism is also explained by the narcissistic need for an attractive and healthy external image for others [37], which could result in better metabolic health. Contrary, findings on psychopathy show the negative effect of this trait on somatic and mental health, which is mostly reattracted to a lack of responsible behavior, engagement in risky activities, and inability to consider negative consequences for one’s own health (e.g., [24]). Due to their decreased perception of fear, individuals high in psychopathy tend to be less scared of diseases and are less likely to adhere to health recommendations [18,39]. However, in individuals with AD, fear and anxiety are more frequent than in healthy populations, which might have led to a lesser extent of psychopathy and, thus, to a more health-adhering behavior. Other factors than narcissism and psychopathy seem to be responsible for the association between AD and MetS, such as lifestyle or medication intake. These aspects, however, may also be influenced by the DT. Further research on the associations between MetS, DT, and AD is recommended to disentangle underlying mechanisms.

Studying the DT traits in individuals with AD in relation to metabolic parameters is important when considering AD as bio-psycho-social disorders since the examination of personality traits within this context allows for a more comprehensive understanding of AD and the underlying mechanisms. AD are typically viewed as complex disorders caused by a combination of biological, psychological, and social factors. Examining the DT traits in conjunction with biological parameters helps to shed light on the biological dimension of AD and how they interact with other factors. In addition, our results contribute to a better understanding of the different manifestations of AD. By considering personality traits, it may be possible to identify subtypes of AD that are linked to specific biological characteristics or pathways, leading to improved diagnoses and new avenues for intervention or treatment, especially concerning the approach of personalized treatment. Further, our results may help individuals with AD and MetS understand the importance of their health behavior and the need to incorporate and maintain healthy lifestyles during therapy to keep up their metabolic health. Considering personality traits like the DT in the treatment of AD could also promote a more individualized therapy and, thus, support faster recovery and better relapse prevention. This includes nutritional counseling, psychoeducation on positive health behaviors, and a possible medical adjustment (e.g., antidiabetics). Our results should raise awareness, especially among general practitioners, internists, and psychiatrists, to take timely preventive measures. Those with higher Machiavellianism should be specifically monitored for metabolic health deteriorations. Our findings serve as an additional aspect in the diagnosis of metabolic health problems in AD and contribute to new treatment considerations for AD.

## 5. Limitations

The results of this study are only applicable to individuals diagnosed with AD. Therefore, follow-up work should include a control group without a psychiatric disorder to investigate whether the effects are AD-specific. In general, DT was in the below-average range in our sample. While this is consistent with other studies indicating that individuals with AD have lower scores in narcissism [28] and some psychopathic traits [26], our results might have been different if individuals scoring high in DT scores would have been included. Further, a conclusion about the causality and direction of our results cannot be drawn due to the cross-sectional study design. Many participants reported an intake of psychotropic drugs at the time of the investigation, which might cause weight gain and could also affect MetS (e.g., [11]). Furthermore, the sample size was rather small and may thus limit generalizability. Therefore, it would be worthwhile to expand the sample size in future studies and reexamine this relationship. It should be noted that this study was not a case–control study but an observational study.

## 6. Conclusions

In summary, the results show no association between MetS criteria and DT in individuals with AD. However, Machiavellianism was positively associated with BMI and negatively associated with systolic blood pressure, indicating that this trait should be closely attended to in individuals with AD to prevent obesity and metabolic comorbidities. Investigating personality traits in somatic conditions of AD could lead to a better understanding and new insights into metabolic disorders in this population. This study thus contributes to the knowledge of factors driving the relationship between MetS and AD and highlights the role of early detection of health-deteriorating aspects in an already vulnerable population.

## Figures and Tables

**Table 1:** Descriptive statistics.

		Metabolic Syndrome			χ^2^/*t*/*U*
Variable		Yes (*n* = 56)	No (*n* = 56)	Total (*n* = 112)	
Age		*M* = 47.70 *SD* = 11.50	*M* = 47.34 *SD* = 11.57	*M* = 47.52 *SD* = 11.49	−0.16
Sex	Female	29 (51.79%)	30 (53.57%)	59 (52.68%)	0.037
	Male	26 (46.43%)	25 (44.64%)	51 (45.54%)	
	Diverse	1 (1.79%)	1 (1.79%)	2 (1.79%)	
Education	Compulsory school	8 (14.29%)	2 (3.57%)	10 (8.92%)	1107.00 **^a^
	Apprentice-ship	31 (55.36%)	26 (46.43%)	57 (50.89%)	
	High school diploma	10 (17.86%)	10 (17.86%)	20 (17.86%)	
	Bachelor diploma	4 (7.14%)	4 (7.14%)	8 (7.14%)	
	Master diploma	3 (5.36%)	12 (21.43%)	15 (13.39%)	
	PhD	0 (0%)	2 (3.57%)	2 (1.79%)	
Somatic disorders		34 (60.71%)	17 (30.36%)	51 (45.54%)	10.40 **
Diabetes mellitus		3 (5.36%)	1 (1.79%)	4 (3.57%)	
Hypertension		19 (33.93%)	8 (14.29%)	27 (24.11%)	
Stroke		3 (5.36%)	1 (1.79%)	4 (3.57%)	
Chronic lung diseases		3 (5.36%)	0 (0%)	3 (2.69%)	
Cancer		3 (5.36%)	1 (1.79%)	4 (3.57%)	
Rheumatic diseases		3 (5.36%)	3 (5.36%)	6 (5.36%)	
Somatic medication		31 (55.36%)	21 (37.50%)	52 (46.43%)	3.59
Psychiatric medication		56 (100%)	56 (100%)	112 (100%)	
Single depressive episode		7 (12.50%)	12 (21.43%)	19 (16.96%)	
Recurrent depression		38 (67.86%)	39 (69.64%)	77 (68.75%)	
Bipolar disorder		8 (14.29%)	6 (10.71%)	14 (12.50%)	
TGL [mg/dL]		*M* = 198.75 *SD* = 128.09	*M* = 111.88 *SD* = 49.09	*M* = 155.31 *SD* = 105.96	−4.74 ***
TGL treatment		9 (16.07%)	4 (7.14%)	13 (11.61%)	2.18
HDL cholesterol [mg/dL]		*M* = 46.00 *SD* = 15.42	*M* = 59.80 *SD* = 18.46	*M* = 52.90 *SD* = 18.29	4.30 ***
Cholesterol treatment		10 (17.86%)	4 (7.14%)	14 (12.50%)	2.94
BP_sys_ [mmHg]		*M* = 131.20	*M* = 124.16	*M* = 127.68	−2.71 **
		*SD* = 14.32	*SD* = 13.10	*SD* = 14.12	
BP_dia_ [mmHg]		*M* = 87.66	*M* = 78.35	*M* = 83.01	−5.17 ***
		*SD* = 9.97	*SD* = 9.07	*SD* = 10.58	
Blood pressure		26 (46.43%)	7 (12.50%)	33 (29.46%)	15.51
treatment					
Fasting Plasma Glucose [mg/dL]		*M* = 100.32 *SD* = 23.47	*M* = 90.80 *SD* = 16.11	*M* = 95.56 *SD* = 20.60	−2.50 **
Diabetes mellitus		5 (8.93%)	1 (1.79%)	6 (5.36%)	
BMI		*M* = 31.03	*M* = 24.38	*M* = 27.70	−7.44 ***
		*SD* = 5.04	*SD* = 4.40	*SD* = 5.77	
Waist circumference [cm]		*M* = 103.68 *SD* = 13.09	*M* = 85.34 *SD* = 11.74	*M* = 94.51 *SD* = 15.42	−7.80 ***

Note: TGL = Triglycerides. HDL = High-Density Lipoprotein. BP_sys_ = Systolic Blood Pressure. BP_dia_ = Diastolic Blood Pressure. BMI = Body Mass Index. ** *p* < 0.01, *** *p* < 0.001. ^a^ Mann–Whitney-*U*-test. Significant results are printed in bold.

**Table 2:** Correlations and significance levels between study variables.

	MetS	DT	Narc	Mach	Psych	Age	Sex
1. MetS	1	0.08	−0.08	0.14	0.11	0.02	0.02
2. DT		1	0.69 **	0.78 **	0.80 **	−0.04	0.32 **
3. Narcissism			1	0.19	0.44 **	0.10	0.36 **
4. Machiavellianism				1	0.47 **	−0.05	0.21
5. Psychopathy					1	−0.16	0.16
6. Age						1	0.27
7. Sex							1

Note: MetS = Metabolic Syndrome. DT = Dark Triad Total Score. Narc = Narcissism. Mach = Machiavellianism. Psych = Psychopathy. ** *p* < 0.01. *n* = 108. Benjamini-Yekutieli adjustments for all *p*-values. Significant results are printed in bold.

**Table 3:** Partial Correlation Analyses (controlling for Sex) and Significance Levels between Study Variables.

	Narc	Mach	Psych	DT	TGL	HDL	BP_sys_	BP_dia_	PG	WC	BMI
1. Narc	1	0.09	0.39 **	0.64 **	−0.14	0.05	−0.06	−0.05	0.19	−0.06	−0.03
2. Mach		1	0.46 *	0.75 **	0.10	−0.19	−0.23 *	−0.05	0.08	0.22	0.29 *
3. Psych			1	0.81 **	0.03	0.03	−0.12	−0.01	0.11	0.18	0.21
4. DT				1	0.00	−0.07	−0.20	−0.05	0.17	0.16	0.22
5. TGL					1	−0.42 **	−0.03	0.05	0.24	0.39 **	0.35 **
6. HDL						1	−0.01	−0.16	−0.02	−0.48 **	−0.59 **
7. BP_sys_							1	0.67 **	−0.03	0.21	0.20
8. BP_dia_								1	0.15	0.32 **	0.32 **
9. PG									1	0.15	0.05
10. WC										1	0.87 **
11. BMI											1

Note: Narc = Narcissism. Mach = Machiavellianism. Psych = Psychopathy. DT = Dark Triad Total Score. TGL = Triglycerides [mg/dL]. HDL = HDL Cholesterol [mg/dL]. BP_sys_ = Systolic Blood Pressure [mmHg]. BP_dia_ = Diastolic Blood Pressure [mmHg]. PG = Plasma Glucose [mg/dL]. WC = Waist Circumference [cm]. BMI = Body Mass Index. * *p* < 0.05, ** *p* < 0.01. *n* = 101. Benjamini-Yekutieli adjustments for all *p*-values. Significant results are printed in bold.

## Data Availability

Data and data codes can be found in the Open Science Frame (OSF) repository, https://doi.org/10.17605/OSF.IO/U9KNS (accessed on 17 August 2023).

## Supplementary Materials

The following supporting information can be downloaded at: https://www.mdpi.com/article/10.3390/metabo13080956/s1, Table S1: Correlations and Significance Levels between Study Variables; Table S2: Partial Correlation Analyses (Controlling for Sex) and Significance Levels between Study Variables.
