# Supplementary material for: Metabolic Syndrome in Affective Disorders: Associations with Dark Triad Personality Traits
Source: Metabolites. 2023 Aug 18;13(8):956. doi: 10.3390/metabo13080956 (PMC10456228; doi:10.3390/metabo13080956)
Supplement: Supplementary file 1 [file metabolites-13-00956-s001.zip › metabolites-2526474-supplementary.pdf]

metabolites

MDPI

Supplementary Materials

Table S1. Correlations and Significance Levels between Study Variables.

	MetS	DT	Narc	Mach	Psych
1. MetS	1	0.12	−0.03	0.13	0.16
2. DT		1	**0.69*** **	**0.74*** **	**0.80*** **
3. Narcissism			1	**0.17***	**0.43*** **
4. Machiavellianism				1	**0.40** **
5. Psychopathy					1

Note: MetS = Metabolic Syndrome. DT = Dark Triad Total Score. Narc = Narcis-sism. Mach = Machiavellianism. Psych = Psychopathy. * $p < 0.05$, ** $p < 0.01$, *** $p < 0.001$. $n = 185$ after exclusion of empty data sets and individuals who identify themselves as diverse gendered. Benjamini-Yekutieli adjustments for all p-values. Significant results are printed in bold.

For the binomial logistic regression analysis, the single DT traits were entered as predictors and MetS as dependent variable. The Hosmer-Lemeshow-Test indicated a good model fit ($\chi^2(8) = 3.06$, $p = 0.931$). The binomial logistic regression model was not statistically significant ($\chi^2(3) = 2.33$, $p = 0.508$, Nagelkerke's $R^2 = 0.02$). Neither narcissism (b = -0.32, $SE = 0.27$, $p = 0.239$; OR[0.43; 1.23] = 0.73), nor Machiavellianism ($b = -0.17$, $SE = 0.22$, $p = .434$; OR[0.77; 1.85] = 1.19), or psychopathy ($b = 0.20$, $SE = 0.29$, $p = 0.489$; OR[0.69; 2.16] = 1.22) predicted MetS. Overall percentage of accuracy in classification was 64.4%, with a sensitivity of 1.5% and a specificity of 99.2%.

Sex and age were included as covariates in the subsequent MANCOVA, which assessed the difference in the single DT traits depending on MetS. Results of the analysis indicated no significant difference between the MetS groups regarding Machiavellianism ($F(1, 0.54) = 0.97$, $p = .327$, $\eta^2_p = .005$), narcissism ($F(1, 0.20) = 0.51$, $p = 0.475$, $\eta^2_p = 0.003$), or psychopathy ($F(1, 0.17) = 0.42$, $p = 0.517$, $\eta^2_p = 0.002$).

Table S2. Partial correlation analyses (controlling for sex) and significance levels between study variables.

	Narc	Mach	Psych	DT	TGL	HDL	BP_{sys}	BP_{dia}	PG	WC	BMI
1. Narc	1	**0.17***	**0.43****	**0.69****	0.06	0.01	−0.11	−0.06	0.04	−0.06	−0.09
2. Mach		1	**0.40***	**0.75****	0.14	−0.07	**−0.18***	−0.08	0.04	0.14	**0.18***
3. Psych			1	**0.80****	0.11	−0.04	−0.09	−0.06	0.06	0.05	0.04
4. DT				1	0.14	−0.05	**−0.18***	−0.09	0.06	0.06	0.07
5. TGL					1	**−0.37****	−0.80	0.05	**0.23****	**0.41****	**0.34****
6. HDL						1	0.27	−0.13	−0.08	**−0.41****	**−0.39****
7. BP_{sys}							1	**0.65****	0.06	**0.24****	**0.28****
8. BP_{dia}								1	0.15	**0.25****	**0.29****
9. PG									1	**0.25****	**0.24****
10. WC										1	**0.89****
11. BMI											1

Note: Narc = Narcissism. Mach = Machiavellianism. Psych = Psychopathy. DT = Dark Triad Total Score. TGL = Triglycerides [mg/dL]. HDL = HDL Cholesterol [mg/dL]. BPsys = Systolic Blood Pressure [mmHg]. BPdia = Diastolic Blood Pressure [mmHg]. PG = Plasma Glucose [mg/dL]. WC = Waist Circumference [cm]. BMI = Body Mass Index. * $p < 0.05$, ** $p < 0.01$. $n = 101$. Benjamini-Yekutieli adjustments for all *p*-values. Significant results are printed in bold.
